# 

**Published:** 1894-06

**Authors:** 


					﻿CONTENTS.
ORIGINAL ARTICLES—
Surgical Shock. By Richard Douglas, M.D., Nashville, Tenn......................... 291
A Case of Gonorrhea in the Female. By R. J. Nunn, M.D., Savannah,
Ga............................................................................. 294
The Extraction of Clear Lenses for Myopia. Report of Five Cases.
By H. F. Stafford, M.D., New York.............................................  296
Operation for Ancient Dislocation of Elbow and Bridge of Callus be-
tween Radius and Ulna. By John B. Roberts, M.D., Philadelphia,
Pa......................................................................................... 299
Suture of the Tendo-Achillis—A Case. By Luther B. Grandy, M.D.,
Atlanta, Ga........................................................................... 301
Cases of Delayed Union in Fracture. By T. G. Morton, M.D., Phila-
delphia, Pa...... ........................................................................ 303
SOCIETY REPORTS—
Clinical Society of Maryland.......................................................... 306
Gynecological and Obstetrical Society of Baltimore........................... 313
The Surgical Section of the College of Physicians of Philadelphia...... 326
BOOK REVIEWS—
Syllabus of the Obstetrical Lectures in the Medical Department of the
University of Pennsylvania. By Richard C. Norris, A.M., M.D.,
Philadelphia, Pa.................................................................. 328
BOOK REVIEWS—Continued—
International Clinics...................................   328.
Antiseptic Therapeutics. By E. L. Trouessart, M.D., Paris, France... 328
Treatment of Typhoid Fever. By D. D. Stewart, M.D......... 328
The Modern Climatic Treatment of Invalids with Pulmonary Con-
sumption in Southern California. By P. C. Remondino, M.D.... 328
SELECTIONS AND ABSTRACTS—
Muscular Rheumatism....................................... 329
Wylie (W. Gill) on Dysmenorrhea and Chronic Endometritis.. 330
Some Therapeutic’ Indications of Codeine..,............... 331
The Treatment of Vesico-Vaginal Fistula................... 332
M elancholia.............................................. 332
Fracture of the Dislocated Humerus....................     333
A New Method of Using Politzer’s Apparatus................ 334
The Justifiable Prevention of Conception.................. 334
Physicians Cannot Testify................................. 335
Pain...................................................... 335
EDITORIAL—
The Congress of American Physicians and Surgeons........... 336
EDITORIAL NOTES............................................... 338
PRESCRIPTION DEPARTMENT..................................  340,	341
Infant Laxative Mixture—Children’s Tonic—Gonorrhea—Anorexia—
Naso-Pharyngeal Catarrh of Infants — Asthma—Saline Laxative—For
Sore Throat.
SPECIAL NOTES .... ................................342,	344, 346, 348
				

